# A Multi-Agent LLM Network for Suggesting and Correcting Human Activity and Posture Annotations

**DOI:** 10.1145/3714394.3756185

**Published:** 2025-12-29

**Authors:** Ha Le, Akshat Choube, Vedant Das Swain, Varun Mishra, Stephen Intille

**Affiliations:** Northeastern University, Boston, MA, USA; Northeastern University, Boston, MA, USA; Tandon School of Engineering, New York University, New York City, NY, USA; Northeastern University, Boston, MA, USA; Northeastern University, Boston, MA, USA

**Keywords:** Large language model, Human postures and activities measurement, Ubiquitous computing

## Abstract

Accurate human activity recognition (HAR) is critical for health monitoring and behavior-aware systems. Developing reliable HAR models, however, requires large, high-quality labeled datasets that are challenging to collect in free-living settings. Although self-reports offer a practical solution for acquiring activity annotations, they are prone to recall biases, missing data, and human errors. Context-assisted recall can help participants remember their activities more accurately by providing visualizations of multiple data streams, but triangulating this information remains a burdensome and cognitively demanding task. In this work, we adapt GLOSS, a multi-agent LLM system that can triangulate self-reports and passive sensing data to assist participants in activity recall and annotation by suggesting the most likely activities. Our results show that GLOSS provides reasonable activity suggestions that align with human recall (63–75% agreement) and even effectively identifies and corrects common human annotation errors. These findings demonstrate the potential of LLM-powered, human-in-the-loop approaches to improve the quality and scalability of activity annotation in real-world HAR studies.

## 1 Introduction

Human activity detection is crucial for enabling context-aware interactive systems, including for health monitoring and interventions. Researchers across ubiquitous computing, human-computer interaction (HCI), digital phenotyping, and behavioral sciences have long sought to use mobile and wearable sensing to develop machine learning models for human activity recognition (HAR) [1, 3, 9, 22, 43]. Developing such models requires large amounts of training data with high-quality labels. In practice, researchers often rely on small, controlled laboratory datasets that offer limited label diversity and overly homogeneous sensor signals. As a result, models trained on these datasets often fail to generalize to free-living contexts, where activities are more heterogeneous, unpredictable, and influenced by individual lifestyle differences [7, 17].

Collecting participants’ self-reports is a practical approach for acquiring multi-day or multi-week activity annotations as individuals go about their daily lives. Researchers can collect self-reports either momentarily or retrospectively. Momentary measurements, such as Ecological Momentary Assessment (EMA) [41], involve prompting participants in real time to report their in-the-moment activity. While effective for capturing immediate behavior, these methods impose an interruption burden and are prone to contextual response biases – participants’ likelihood of responding depends heavily on their environment and situation at the time of the prompt. This often leads to data missingness and label imbalance, thus compromising the quality of the dataset [27, 34, 39].

Retrospective self-reports mitigate these issues by allowing participants to recall and report their activities after-the-fact, typically at the end of the day [1]. While less intrusive, retrospective recall is cognitively demanding and vulnerable to recall biases, as prior or subsequent events could distort memory accuracy. Two common errors in retrospective activity recall are: 1) temporal errors, where participants misremember the start or end time of events; and 2) omission of concurrent activities, where secondary activities are forgotten. To reduce temporal errors, researchers have developed automated, context-assisted recall tools that provide participants with contextual cues – such as location data or sensor-derived summaries – to help participants reconstruct their daily activities [40, 44]. Nevertheless, for participants in research studies, who often have limited time, patience, and cognitive resources, reviewing and triangulating multiple sources of data for accurate recall is a significant burden [26].

Recent advancements in large language models (LLMs) present a promising opportunity for HAR [4, 38]. LLMs possess broad commonsense knowledge and strong contextual reasoning abilities, enabling them to integrate and triangulate information from multiple heterogeneous sources (e.g., location traces, wearable sensor data, calendar events, and environmental context) to infer likely activities and patterns [5, 45]. We argue that LLMs can proactively suggest likely activities, flagging potential annotation inconsistencies, and reducing the cognitive burden on participants during the recall process. In this work, we adapt GLOSS, a multi-agent LLM system originally designed for general contextual reasoning of passive sensing data [5], to the problem of HAR, demonstrating its potential to serve as a core component of an intelligent, context-assisted, activity annotation framework.

In this paper, we make the following key contributions:

- We present an extension of GLOSS, a multi-agent LLM framework, applied to the problem of human posture and activity annotation. Our findings suggest that GLOSS’s activity annotations aligned with participant recall (63–75%), indicating its potential to suggest activities.
- Through qualitative analysis, we show that several discrepancies between GLOSS predictions and participant annotations may stem from human recall limitations. We provide examples where GLOSS highlights inconsistencies that could help identify and correct recall-related errors.
- We position this work as a proof of concept, demonstrating the feasibility of leveraging LLMs to support human-in-the-loop activity annotation. We also discuss future directions to expand the framework’s capabilities and validate its utility over longer-term, multi-day deployments.

## 2 Background

We build our work upon prior literature in human activity recognition (HAR) and recent works on integrating large language models (LLMs) with passive sensing data from phones and wearables.

### 2.1 Measuring human postures and activities

Human activity recognition (HAR) involves identifying specific activities or postures from sensing data, with accurate models critical for powering interactive systems and understanding daily behaviors. Building such models demands labeled data, but most existing HAR datasets, which researchers collected in controlled environments with limited labels, do not capture the complexity and variability of real-world behavior [3, 7, 17]. As a result, these models often generalize poorly in free-living settings, where activities are subtle, overlapping, and diverse, and they struggle to adapt to new sensors or unseen activities without retraining [19]. Researchers have explored simulating labeled free-living data using video or language approaches [24, 28], and applying self-supervised learning by pretraining on large unlabeled datasets before fine-tuning on labeled data [15, 16, 19].

Participant self-reports offer another scalable way to gather multi-day labeled data [2, 47], though they remain prone to temporal misalignment, missing labels, and recall biases that can degrade model quality [23]. These limitations highlight the need for human-in-the-loop systems that can intelligently suggest and refine activity annotations, improving both the scalability and accuracy of data collection—and ultimately enabling more robust, adaptable HAR models for real-world use.

### 2.2 LLMs with passive sensing data

LLMs have shown potential in understanding and predicting health and well-being outcomes (like depression, stress, and activities [21, 38, 48]) from passive sensing data from smartphones and wearables. As LLMs understand natural language better than a long sequence of numbers, a common approach is to convert sensor data into natural language formats, enabling LLMs to make predictions about health outcomes [31, 46]. Additionally, researchers have leveraged LLMs to generate narratives and summaries of passive sensing data for users, showing that such formats can enhance reflection and engagement [11, 29, 35, 45]. Some prior works have also fine-tuned LLMs on sensor data for different tasks like sensor-to-text conversions[4] and drawing health insights[8]. As fine-tuning requires time and effort, more recently, researchers have started looking at multi-agent systems to build zero-shot models to derive insights from passive sensing data[5, 33]. Choube et al. [5] developed GLOSS, an open-ended multi-agent sensemaking system for passive sensing data. GLOSS is an easy-to-deploy task-based system capable of triangulating multi-modal data and presenting insights tailored to the needs of the user. In this work, we extend GLOSS for the task of triangulating multi-modal sensor streams to generate suggestions for human activity annotations and also correcting annotation errors and inconsistencies.

## 3 Methodology

We extend GLOSS and compare the system’s ability to generate activity annotations by triangulating passive sensing information to participants’ self-reported activity annotations in a research study.

### 3.1 ACAI platform and dataset

**Figure 1:**
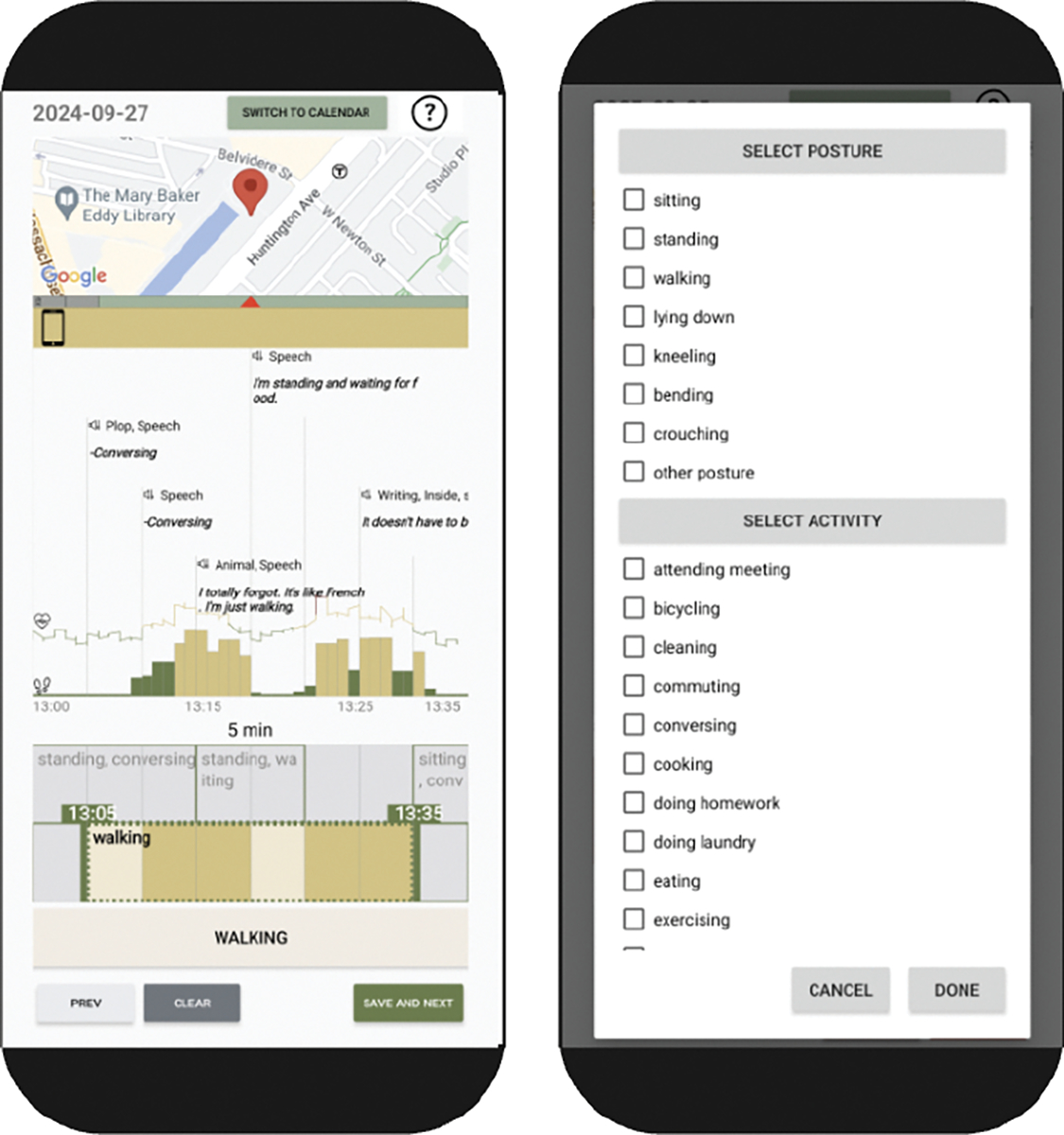
Screenshots of the ACAI annotation app, where participants can review their passive sensing data and annotate their posture/activities throughout their waking day.

ACAI (**A**Ctivity **A**nnotation **I**nterface) is a mobile app for research data collection, capturing both passive sensing data and participant-provided activity annotations (Figure 1) [26]. Eleven participants wore a Pixel 2 smartwatch that recorded passive sensor data and *μ*EMA in-the-moment activity self-reports [27]. After two days of data collection, they participated in a one-hour session using the ACAI app to annotate their activities from the previous day. The app displayed visualizations of the collected sensing data and *μ*EMA responses to help participants recall and label their activities accurately. Every 15 minutes, participants answered *μ*EMA prompts on the smartwatch via speech [25, 27]. The app transcribed speech on-device using a fine-tuned Google Cloud Speech-to-Text model [13] and saved only the transcriptions. The list of passive sensing data and self-reports displayed on the mobile app for participants and available in the dataset is Table 1.

Validation studies of ACAI showed that while context-assisted and heuristic-based segmentation reduces participant burden and improves annotation accuracy compared to 24PAR and ACT24 [20, 49], the resulting labels still contain errors, including inaccurate boundaries, missing secondary activities, and overlooked short bursts of activity. These issues stem not only from the cognitive effort of integrating multiple data sources but also from human factors constraints like inattention and reluctance to create detailed labels. These issues extend beyond the ACAI platform and are present in many other self-reflection systems [32, 47]. Leveraging LLMs can help address these challenges by suggesting likely activities and postures based on passive sensing data, providing supporting evidence, and flagging inconsistencies to guide more focused human feedback.

### 3.2 GLOSS: System overview

GLOSS is a system consisting of multiple LLM agents designed to mimic the process of sensemaking in humans. This sensemaking process in GLOSS involves two cyclic processes: the *Information seeking* phase focusing on retrieving information from the datasets and processing the raw data into more a understandable format; and the *Sensemaking* phase focusing on triangulating and interpreting the results from multiple data streams, as well as presenting the final results.

**Figure 2:**
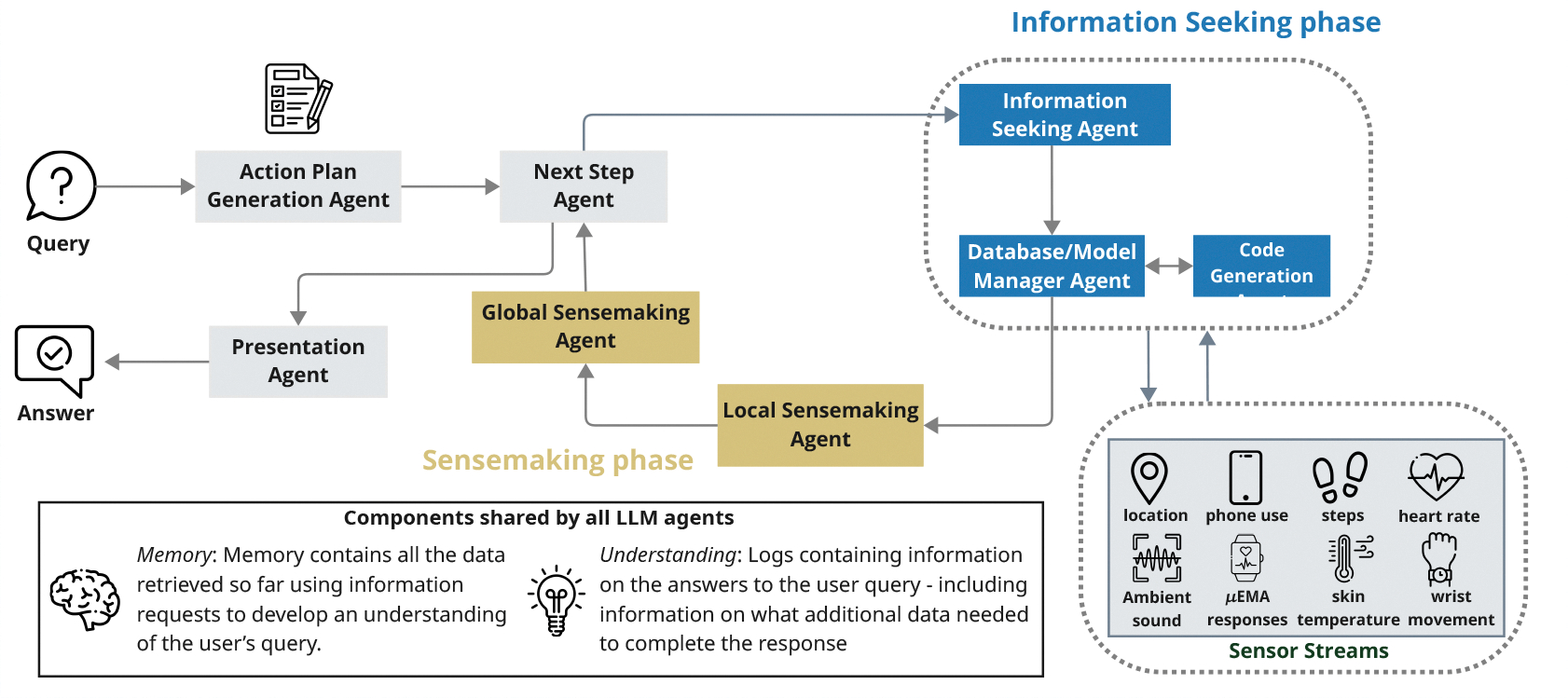
Networks of LLM agents and their interactions in GLOSS.

GLOSS’s network includes eight LLM agents (Figure 2):

- ***Action plan generation agent***: This agent takes in the user query and creates a high-level plan to answer the user query using the available data.
- ***Next step agent*:** In each cycle, the *Next step* agent determines whether the current *understanding* sufficiently answers the user query based on the *action plan*. If not, the agent enters the information-seeking-sensemaking loop.
- ***Information seeking agent*:** This agent creates specific information requests to retrieve information from the databases to answer the user query. This can involve fetching, processing, or triangulating multiple data streams.
- ***Database manager agent* and *Code generation agent*:** The *Information seeking agent* can pass requests to *Database manager agent* to retrieve or process data. Using some pre-defined helper functions, the *Code generation agent* writes and executes Python scripts to process the data. The final results are sent to the *Sensemaking* loop.
- ***Local and global sensemaking agents*:** The results of data retrieval and code execution process are passed to the *Local sensemaking agent* to generate a natural language representation. The system adds these results, along with the *information requests* generated by the *Information seeking agent*, to the *memory*. The *Global sensemaking agent* then reviews the *action plan*, *previous understanding* and *memory* to create a new *understanding* of the user query. The process then goes back to the *Next step agent*, completing one iteration of the *sensemaking* loop.
- ***Presentation agent*:** Once the *Next step agent* determines that the current *understanding* is sufficient for the user query, it hands the process over to the *Presentation agent*. The agent extracts the response to the user query from the *understanding*, and formats the response according to the user-specific presentation instructions (if applicable).

### 3.3 Adapting GLOSS for Suggesting and Correcting Annotations

The original GLOSS design focused on creating a query-based system with a minimal learning curve, enabling users to ask about a wide range of topics—such as stress, mobility, or social interactions—through a chat interface. In this work, we introduced several design modifications (DMs) to better support suggestions and corrections for human posture and activity annotations.

#### DM1: Emphasis on change detection helper functions to identify start and stop time of activities.

The GLOSS system allows adding helper functions to assist in processing data and performing triangulation. In this work, we focus on the task of activity suggestion, which involves two sub-tasks: (1) identifying the start and stop times of activities, and (2) identifying the activity labels. Although LLMs are capable of using contextual information and self-reports to infer activity and posture labels, they struggle with understanding temporal structures. Tasks like change-point detection from raw data require logical and numerical reasoning, which LLMs often fail to provide [42]. To address this, we extended GLOSS with pre-defined change point detection functions for each data stream to process transitions better. For step count, heart rate, skin temperature, and wrist AUC data, we implemented a heuristic sliding window z-score–based change point detection algorithm with a minimum segment duration of 60 seconds [12]. For location data, we used DBSCAN to identify clusters where participants spent a significant amount of time [10]. For phone usage, we provided a pre-defined function that extracts periods of continuous phone interaction.

#### DM2: Effects of detection windows on activity agreement with human annotations.

We passed the list of data collected by ACAI (Section 3.1) to GLOSS in fixed-length segments – for example, asking it to suggest a list of postures and activities a participant engaged in from 8am to 9am (one-hour window), or from 8am to 10am (two-hour window). This method keeps the input within the language model’s context length and fits well in real-time systems. This approach, however, has two drawbacks. GLOSS may lose continuity between time windows, which increases the number of tokens it needs to generate, since it must recreate action plans and code from scratch each time. It can also lead to inconsistent responses, especially for longer activities that span multiple windows. To address these issues, we adopted a *temporally-persistent* implementation strategy, where we freeze the same *action plan* across consecutive time windows. Additionally, we pass *understanding* of the previous time window as an input to the sensemaking process of the current time window. This approach allows GLOSS to behave more like a cohesive system that builds on prior context, rather than treating each prompt as an isolated task.

#### DM3: Consistent presentation of results and mapping of postures and activities.

GLOSS was originally designed as an open-ended query interface. In the context of human activity recognition and health sciences, however, researchers are interested in a defined and structured set of labels. Thus, we want GLOSS to generate labels from a predefined set of relevant activities, rather than producing arbitrary ones. To ensure consistency and robustness, we provide the list of predefined posture and activity labels (Table 2) – along with the desired output format, to the *Presentation agent*. Our design aims to support the integration of our system to more structured activity recognition or intervention systems that require fixed format. A structured output also makes the validation process easier. The list of activity labels, however, can be extended or made open-ended based on the requirements of the study.

**Figure 3:**
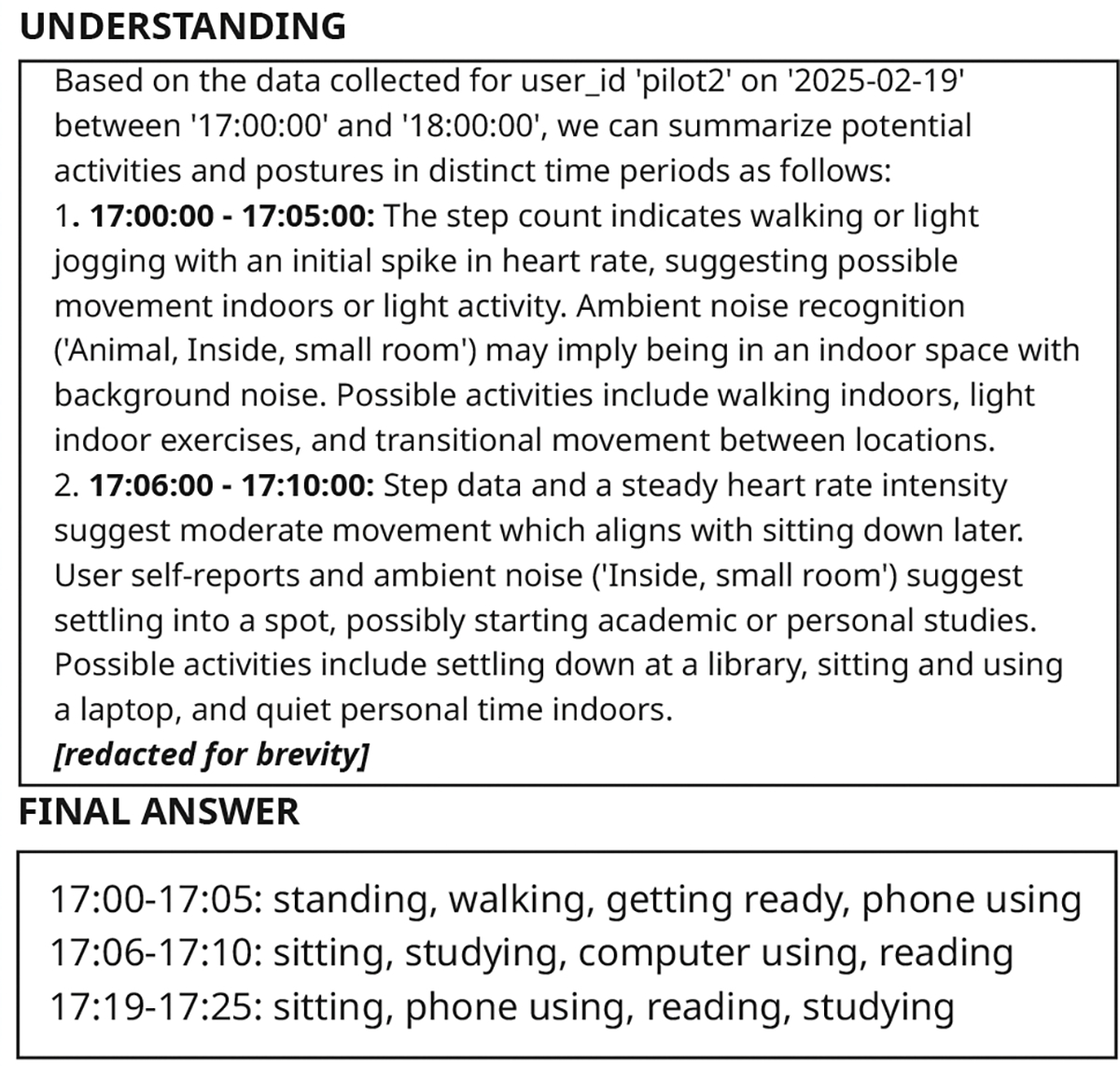
Example understanding and final answer generated by GLOSS.

The GLOSS framework is implemented in Python using the LangChain and AutoGen frameworks to manage interactions between LLM agents. We used GPT-4o [37] as our Large Language Model and designed prompts following OpenAI’s recommended best practices for prompt engineering [36]. In our GPT-4o API calls, we set *temperature* = 0 and *top*_*p*_ = 1. To ensure security and prevent potential harm to the system running GLOSS, we executed any code generated by the LLM models within a Docker container. In our experiment, we stored the raw passive sensing data from the ACAI dataset in MongoDB databases and connected these to the *Database Management Agent* within GLOSS. We show an example of running GLOSS with detection window of one hour for a participant in Figure 3.

## 4 Results

In this section, we present the results of running GLOSS through the dataset annotated by the participants in the ACAI study.

### 4.1 Comparison with human recall

We present the agreement rate between participants’ self-annotations with GLOSS suggestions in Table 3. We calculate the agreement rate using the following formula:

$$AgreementRate=\frac{\#\mathrm{labelsSuggested}}{\#\mathrm{totalLabels}}$$

where #labelsSuggested is the number of labels annotated by the participants that are also in the list of labels suggested by GLOSS, and #totalLabels is the total number of labels annotated by the participants. We do not account for false positives produced by GLOSS in our metric, as the intended application of GLOSS is to serve as a suggestion tool for activity annotation. Since GLOSS can suggest three activities and one posture at a time, its high positive rate means that, even in the presence of some false positives, it might still reduce the annotation effort on participants.

GLOSS predictions show good agreement with human annotations at smaller time windows of 30 minutes and one hour but exhibit lower agreement at longer windows of two hours or more. Although we configured GLOSS to produce suggestions at minute-level granularity, its outputs often default to fixed-length time blocks (such as 10, 15, or 30 minutes) when using longer detection windows. In the example below, in a one-hour window, GLOSS generated dynamic, minute-by-minute suggestions. In contrast, with a two-hour window, it segmented the timeline into uniform 15-minute blocks.


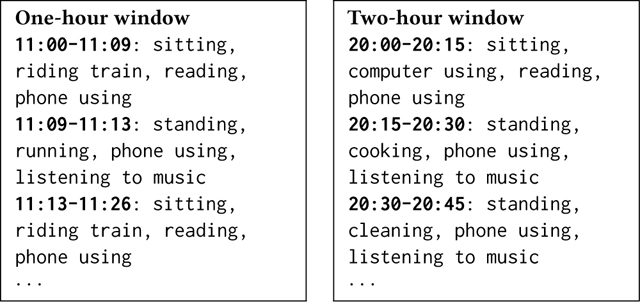


### 4.2 Potential to fix incorrect annotations

In our experiments and quantitative evaluations, we treat human annotations as the best approximation of ground truth for postures and activities. This is based on the premise that participants could review multiple data sources and annotate using both the data and their memory. These annotations, however, are still susceptible to error. Due to limited screen space in the phone, cognitive and time demands, participants may overlook brief events or struggle to triangulate information from multiple data streams. Given such inconsistencies, GLOSS can be especially valuable in a human-in-the-loop annotation workflow, where it can help *flag inconsistencies* or even *correct potential errors* in the participants’ labels. Based on our analysis, we found three common types of annotation mistakes: participants often 1) failed to annotate ***short bursts of activity***; 2) ***omitted secondary activities*** when multitasking; and 3) ***made mistakes in the start/stop time (temporal errors)***.

**Figure 4:**
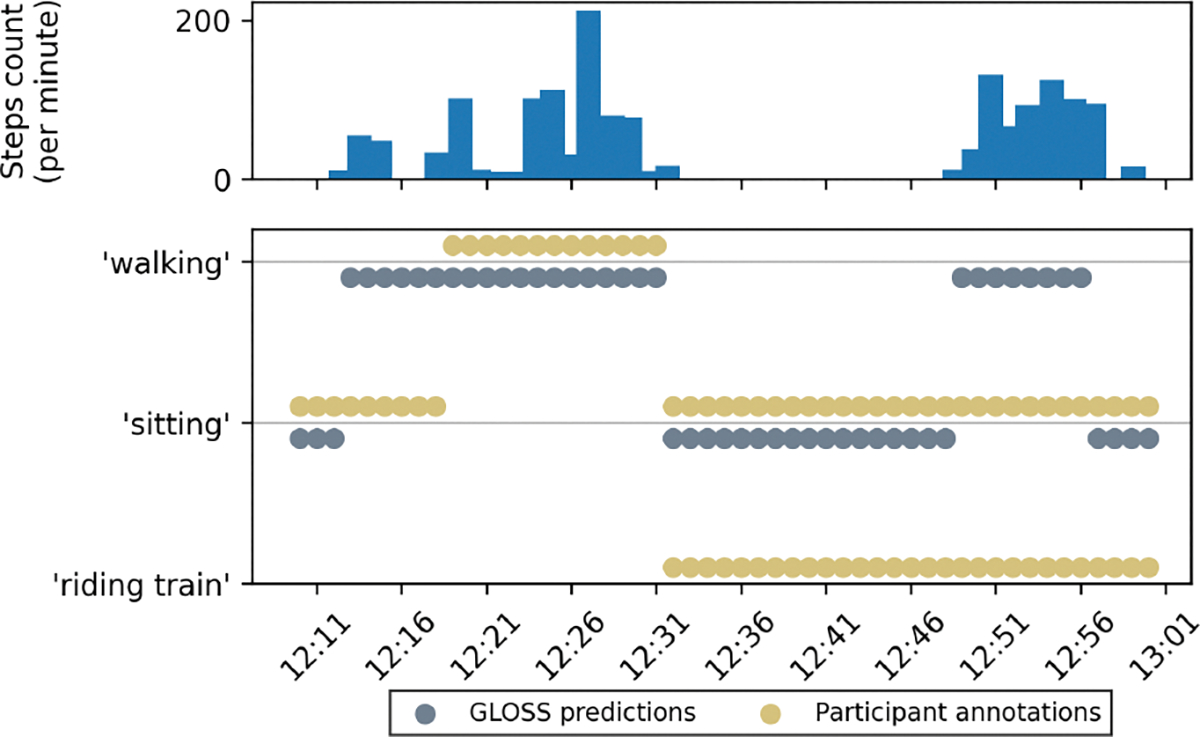
GLOSS flagged potential missing label

We show an example of the first type of mistake in Figure 4. In this example, the participant annotated ‘sitting, riding train’ from 12:30 p.m. til 1 p.m. The step count from Pixel watch, however, indicates that there was a brief period of ‘walking’ from 12:47pm to 12:55pm. GLOSS was able to flag this period as ‘walking’.

**Figure 5:**
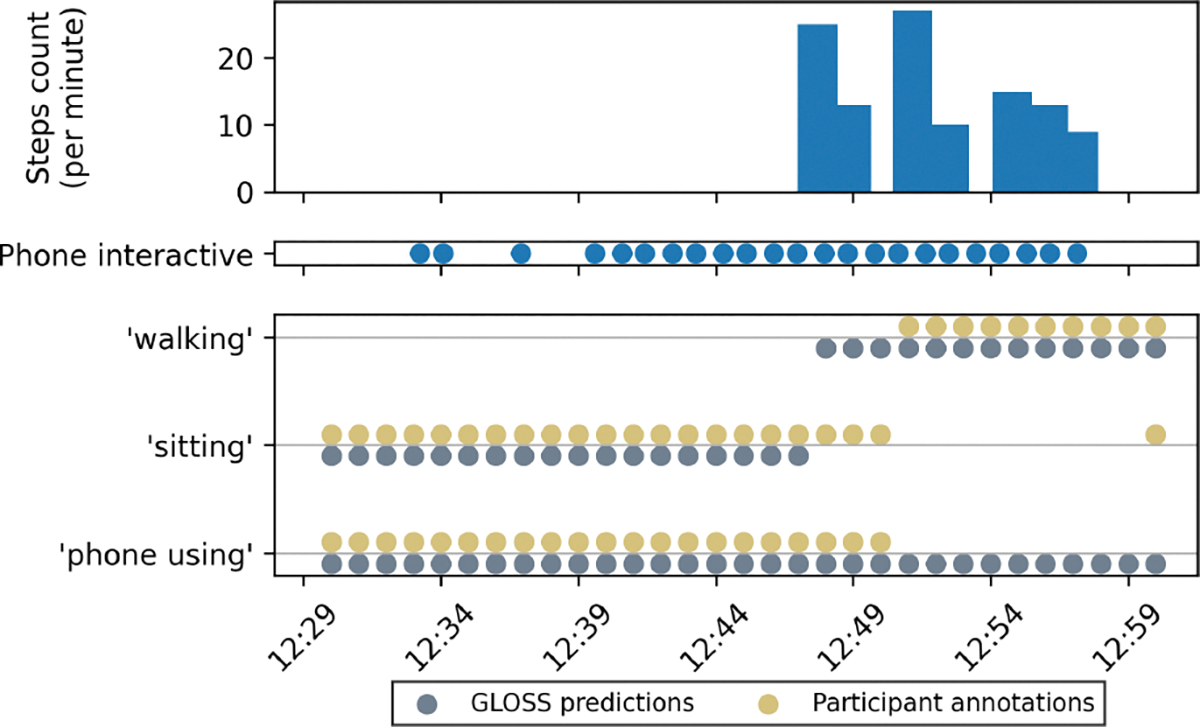
GLOSS added labels for secondary activity.

We present another example of GLOSS correcting participants’ annotations in Figure 5. The participant was using phone between 12:30pm-1pm. There was, however, a change of activity from ‘sitting’ to ‘walking’ around 12:50pm. We believe that since the participant viewed ‘walking’ as their main activity, they forgot to include ‘using phone’ as a secondary activity. Using the participant’s phone usage data, GLOSS was able to flag ‘using phone’ as the secondary activity label, providing more information to the participant’s contextual states and behaviors.

In both examples (Figure 4 and 5), the participants underestimated the start time of the ‘walking’ label. In Figure 4, the first bout of ‘walking’ began around 12:12, but the participant annotated the start time as 12:18. Similarly, in Figure 5, the participant started walking around 12:47, but the start time was annotated as 12:50. GLOSS was able to cross-reference with the step count to fix the boundary of the label. Although these mistakes may seem minor, prior work has shown that even small temporal misalignments can reduce the performance of HAR models trained on such data [23].

## 5 Discussions and Future Works

In this section, we discuss our results, outline the potential of a multi-agent LLM system for human activity annotation, and reflect on the implications and limitations of our work.

Recalling activity is a time-consuming and cognitively demanding task for participants. *μ*EMA —a method where individuals report their current activity and posture via their phone or smartwatch—offers a promising approach to support later recall during annotation[18]. A key limitation of *μ*EMA is the frequency of prompts: prompting frequently can become burdensome and even frustrating for participants, ultimately affecting their compliance. Human-in-the-loop systems that combine users’ self-reports (*μ*EMA) with passive sensing data from phones and wearables to improve recall quality offer promising direction. In this work, we take a step forward in human-in-the-loop systems by extending a LLM-based system GLOSS for suggesting and correcting activity annotations using participants’ *μ*EMA responses and passive sensing data.

**Figure 6:**
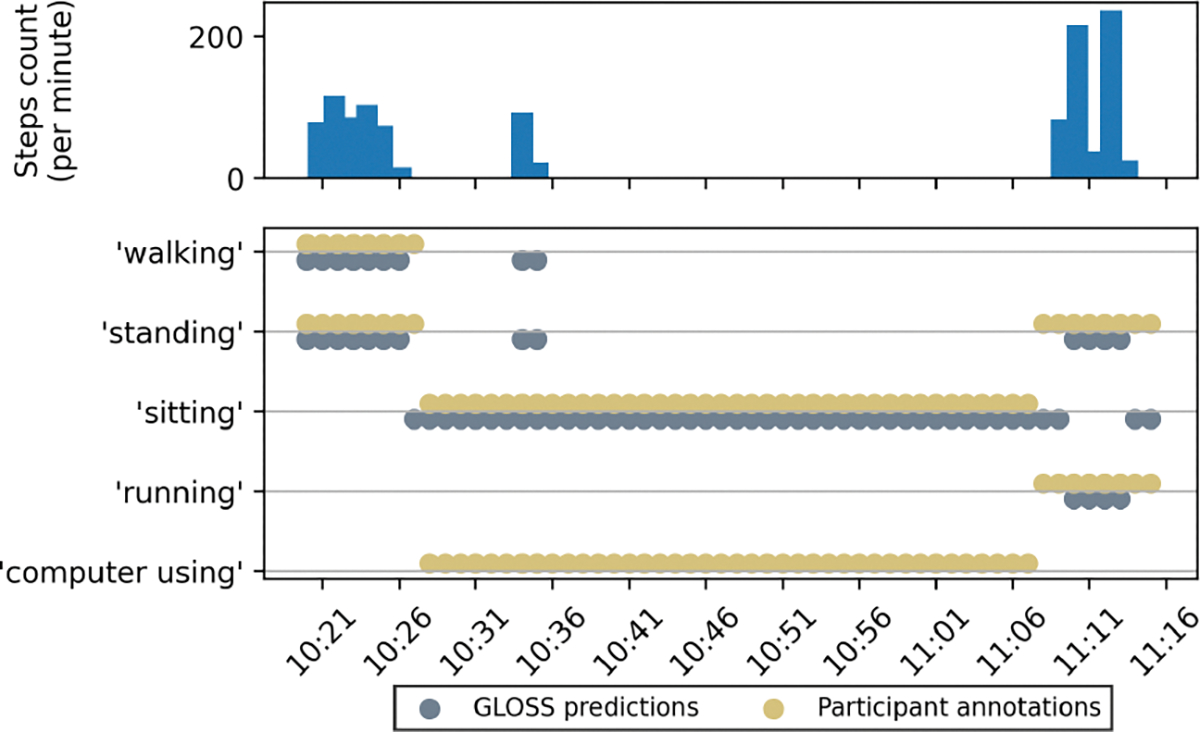
GLOSS failed to suggest the correct activity without the appropriate data and context (no data from computer).

The quantitative and qualitative results from our preliminary experiments show positive signal for assisting activity annotation. For participants, reliable suggestions integrated into the annotation interface means they might not need to browse through an exhaustive list of activity labels. For researchers, our system can help correct inaccurate annotations, reducing the need for manual data cleaning when building activity recognition models. While these are some direct implications, we also believe that our system has tremendous potential in longitudinal free-living studies. In such settings, our system may initially rely on participant input (e.g., via *μ*EMA or recall) to learn their routines (Figure 6). Over time, as it gathers more passive sensing data, the system could begin to infer daily activities autonomously, prompting participants only when irregularities or uncertainties are detected. We do not envision a system that removes participant input entirely; instead, we see an evolving system that intelligently balances automation with selective user engagement, reducing burden while preserving accuracy [6, 30].

Despite these promising directions, our current work has limitations. Due to the scope of this workshop paper, we did not conduct a comprehensive quantitative evaluation of all aspects of GLOSS. Additionally, our evaluation was based on a single day of participant annotations, which limits the generalizability of our findings. In future work, we plan to categorize different types of annotation errors and expand our system to support real-time flagging and correction of participant-generated annotations. We also plan to explore the integration of GLOSS with existing annotation interfaces to better support both structured and open-ended activity sensemaking.

## Figures and Tables

**Table 1:** Data streams and their sampling frequencies

Device	Data Stream (Sampling Frequency)
**Phone**	GPS location (1m), app use (1m), lock/unlock events (1m)
**Smartwatch**	Step counts (1m), ambient noise classification [14] (5m), heart rate (1m), *μ*EMA responses (15m), skin temperature (10s), wrist movement data (10s).

**Table 2:** List of postures and activities passed to the *Presentation agent* for generating predictions.

Postures	Activities
sitting, standing, lying down, reclining, upright	video gaming, walking, stair climbing, getting ready, driving, bicycling, vigorous bicycling, aerobics, cleaning, cooking, laundry, playing with pet, listening to music, watching movies/TV, studying, reading, riding in car, riding train, riding bus, playing musical instruments, attending meeting, computer using, phone using, running, getting dressed, grooming, using bathroom, eating, talking, strength training, washing dishes, carrying groceries, putting away groceries, shopping, making bed, packing/unpacking, sleeping, playing sports

**Table 3:** Agreement rate between GLOSS predictions and participants’ self-annotated postures and activities.

Window size	Agreement rate

30-minute	0.73 (*SD* = 0.10)
One-hour	**0.75 (*SD* = 0.11)**
Two-hour	0.63 (*SD* = 0.13)
Four-hour	0.65 (*SD* = 0.14)

## CCS CONCEPTS

• Human-centered computing → Ubiquitous and mobile computing design and evaluation methods.
